# Computational modeling suggests distinct, location-specific function of norepinephrine in olfactory bulb and piriform cortex

**DOI:** 10.3389/fncom.2015.00073

**Published:** 2015-06-16

**Authors:** Licurgo de Almeida, Seungdo J. Reiner, Matthew Ennis, Christiane Linster

**Affiliations:** Computational Physiology Lab, Department of Neurobiology and Behavior, Cornell UniversityIthaca, NY, USA

**Keywords:** noradrenaline, olfactory bulb, piriform cortex, odor detection, odor learning, odor recall

## Abstract

Noradrenergic modulation from the locus coerulus is often associated with the regulation of sensory signal-to-noise ratio. In the olfactory system, noradrenergic modulation affects both bulbar and cortical processing, and has been shown to modulate the detection of low concentration stimuli. We here implemented a computational model of the olfactory bulb and piriform cortex, based on known experimental results, to explore how noradrenergic modulation in the olfactory bulb and piriform cortex interact to regulate odor processing. We show that as predicted by behavioral experiments in our lab, norepinephrine can play a critical role in modulating the detection and associative learning of very low odor concentrations. Our simulations show that bulbar norepinephrine serves to pre-process odor representations to facilitate cortical learning, but not recall. We observe the typical non-uniform dose—response functions described for norepinephrine modulation and show that these are imposed mainly by bulbar, but not cortical processing.

## Introduction

Both the olfactory bulb (OB) and the piriform cortex (PC) are innervated by the locus coeruleus (LC), which releases the catecholamine neuromodulator norepinephrine (NE). NE plays a critical role in regulating vigilance and modulating responses to stimuli that are novel or salient (Foote et al., 1980; Vankov et al., 1995). Work in somatosensory, auditory, olfactory, and visual processing areas showed that NE modulates neuronal responses to sensory stimuli in many modalities and has been shown to convert subthreshold inputs to suprathreshold responses. NE also reduces spontaneous, but not stimulus evoked firing, thereby increasing signal-to-noise ratios (Waterhouse et al., 1990; Mouradian et al., 1991; McLean and Waterhouse, 1994; Devilbiss and Waterhouse, 2004; Devilbiss et al., 2006; Hirata et al., 2006). In the olfactory system, NE has a potentiating effect on weak sensory inputs similar to its effect in other sensory systems (Jiang et al., 1996; Ciombor et al., 1999; Hayar et al., 2001; Bouret and Sara, 2002). The OB and PC are tightly interconnected, receive common neuromodulatory inputs and hence present a unique opportunity to ask how preprocessing (OB) and cortical processing (PC) are modulated in concert to regulate sensory function.

We here present a computational model of OB and PC (de Almeida et al., 2013; Devore et al., 2014) in which NE modulation has been implemented to study how NE modulation in both structures interact to modulate olfactory perception and learning. Processing in the olfactory bulb has classically been assumed to prepare and shape odor representations to be learned in olfactory cortical areas (see Cleland and Linster, 2005 for review). Odor representations in the olfactory bulb are modulated by olfactory learning (Mandairon and Linster, 2009), both with respect to mitral cell firing rates (Kay and Laurent, 1999; Doucette and Restrepo, 2008), oscillatory dynamics (reviewed in Kay et al., 2009), and synchronization properties (Doucette et al., 2011). This type of plasticity can be assumed to be heavily dependent on neuromodulatory inputs, including those from the LC (Doucette et al., 2011). Odor representations in olfactory cortices are also modulated through olfactory learning, and change as a function of odor valence and reward expectation (Calu et al., 2007; Roesch et al., 2007; Gire et al., 2013). The piriform cortex has long been proposed to act as an associative memory network in which odor representations can be stored in synapses between mutually connected pyramidal cells (Haberly and Bower, 1989). Recent experimental work supports this hypothesis, showing pattern completion (Wilson, 2009) and prototype representation formation (Shakhawat et al., 2014a,b) in response to odor learning. Brain slice physiology paired with computational modeling work supports the idea that NE modulation in the olfactory cortex can modulate cortical associative memory function (Hasselmo et al., 1997) by regulating signal to noise ratios as well as synaptic transmission between pyramidal cells. Hence, NE in both the OB and cortex modulates olfactory learning, yet the interaction between these two structures and coordination of neuromodulation has not been studied.

Using a computational approach, we here show that NE differentially modulates odor representations in the OB and PC. At low odor concentrations, bulbar OB enhances the detection of odorants, as shown experimentally (Escanilla et al., 2010). While cortical odor responses are dominated by bulbar inputs in a naïve network, after learning, they are driven mostly by intrinsic cortical synapses. Cortical NE strongly modulates odor learning but can impair recall. In summary, we find that during detection of novel odors, bulbar NE enhances performance. Cortical and bulbar NE cooperate to enhance the learning of odorants in the cortical network, however, NE impairs the recall of learned odor patterns in cortex.

## Methods

### Network architecture

**Figure 1:**
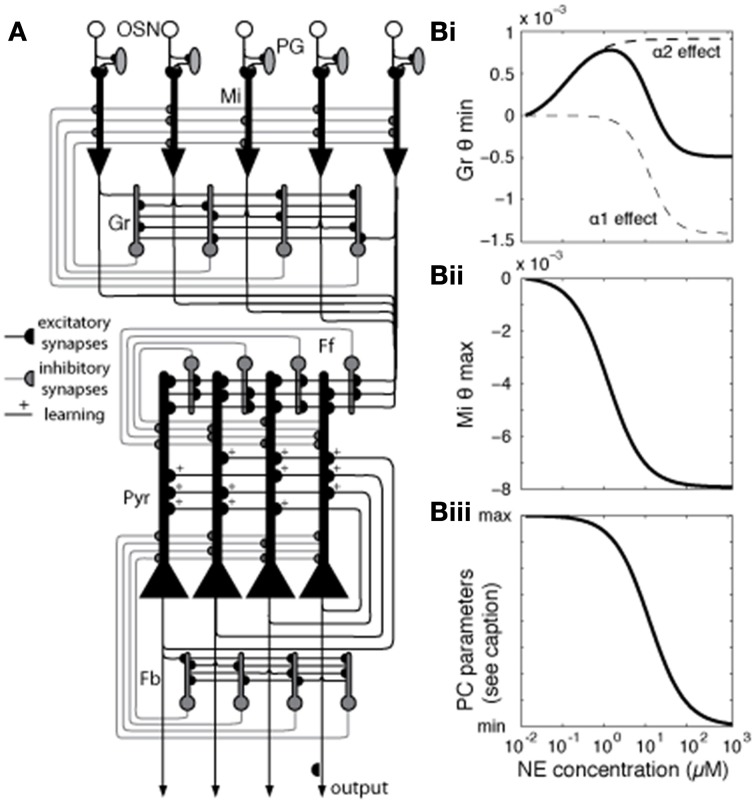
**Network architecture and effects of NE modulation on different mode parameters**. **(A)** Generalized network structure. OSNs that express a common receptor type project to a specific glomerulus, feeding into mitral (Mi), and periglomerular (PG) cells in that glomerulus. Mi cells are the principal output neurons of the OB and are modulated by PG and granule (Gr) inhibitory interneurons. In our model, each PG cell directly inhibits the mitral cell primary dendrite associated with the same glomerulus. Gr cells connect with Mi cells via reciprocal dendodendritic synapses, where Mi-Gr synapses are excitatory and Gr-Mi synapses are inhibitory. Each mitral cell is randomly connected to 40% of the granule cells, without any topological organization. Mi axons project to the PC, forming excitatory connections with pyramidal (Pyr) and feedforward (Ff) inhibitory interneurons which connect to apical dendrites of Pyr cells (30% connectivity), modulating bulbar input (Stokes and Isaacson, 2010), with each pyramidal cell receiving input from 20%, and each Ff interneuron from 40% of mitral cells. Pyr cells form autoassociative connections with 20% other Pyr cells as well as projecting to additional inhibitory interneurons, feedback (Fb) cells (Marr, 1971; Stokes and Isaacson, 2010). **(B)** Modulation of neural parameters by NE in the model [see Equation (1) for details]. In our model, NE affects Mi and Gr cells in the OB and Pyr and Fb cells in the PC. The graphs show how parameters in the model behave (y-axis) for different levels of NE concentration (x-axis). **(B_i_)** shows how NE concentration affects Gr spontaneous firing by changing the spiking threshold (θ_min_) via α1 and α2 receptors (Nai et al., 2010). These receptors modulate Gr spontaneous activity in opposite directions, each with its own dose-response curve. Spontaneous firing increases as θ_min_ increases. The graph in **(B_ii_)** shows how NE changes the excitability of Mi cells by modulation the saturation threshold θ_max_. Excitability, measured as responses to very low inputs, increases as θ_max_ decreases. Finally, the graph in **(B_iii_)** shows how NE modulation changes parameters of Pyr and Fb cells as well as synaptic parameters in PC. PC parameters modulated with the dose response curve shown are excitatory synaptic transmission (reduced from 100 to 60%), the amplitude of the hyperpolarization current [Equation (5)], and qmin in Fb interneurons [Equation (3)].

The model presented here has been adapted from previous work (de Almeida et al., 2013; Devore et al., 2014) to simulate experimentally described effects of NE modulation in the OB and PC. Briefly, the OB and PC are each implemented in separate subnetworks. Four cell types make up the OB: olfactory sensory neurons (OSNs), mitral (Mi) cells, periglomeular (PG) cells, and granule (Gr) cells, connected as shown in Figure 1A, with details as described in de Almeida et al. (2013) and parameters listed in Table 1. The OB model contains 100 neurons of each cell type.

**Table 1:** **Model parameters**.

General parameters (all neurons)	*v*^*hyper*^ = −10 mV; *t*^*refrac*^ = 2 ms^*^.
Olfactory Sensory Neuron (OSN)	τ = 5 ms; β = 1; θ^min^ = 0 mV; θ^max^ = 15 mV.
Mitral (Mi, apical compartment)^‡^	τ = 4 ms.
Mitral (Mi, soma compartment)^‡^	τ = 5 ms; β = 2; θ^min^ = −1.4 mV; θ^max^ = 9 mV/1 mV^†^
Periglomerular (PG)	τ = 2 ms; β = 1; θ^min^ = 0 mV; θ^max^ = 4 mV
Granule (Gr)	τ = 5 ms; β = 2; θ^min^ α1 = −1 mV/–2.4 mV^†^; θ^min^ α2 = 0 mV/1 mV^†^; θ^*max*^ = 6 mV
Feedforward (Ff)	τ = 5 ms; β = 1; θ^min^ = 0 mV; θ^max^ = 15 mV.
Pyramidal (Pyr)	τ = 10 ms; β = 2; θ^min^ = 0 mV; θ^max^ = 15 mV.
Feedback (Fb)	τ = 5 ms; β = 2; θ^min^ = 0 mV/–0.1 mV^†^; θ^max^ = 15 mV
OSN to PG	g^max^ = 0.166; E_N_ = +70 mV; τ_1_ = 1 ms; τ_2_ = 2 ms
OSN to Mi (apical)	g^max^ = 0.16; E_N_ = +70 mV; τ_1_ = 1 ms; τ_2_ = 2 ms
PG to Mi (apical)	g^max^ = 0.38; E_N_ = −10 mV; τ_1_ = 4 ms; τ_2_ = 8 ms
Mi (soma) to Gr	g^max^ = 0.02; E_N_ = +70 mV; τ_1_ = 1 ms; τ_2_ = 2 ms
Gr to Mi (soma)	g^max^ = 0.18; E_N_ = −10 mV; τ_1_ = 4 ms; τ_2_ = 8 ms
Mi (soma) to Ff	g^max^ = 0.2; E_N_ = −10 mV; τ_1_ = 4 ms; τ_2_ = 8 ms
Mi (soma) to Pyr	g^max^ = 0.76; E_N_ = +70 mV; τ_1_ = 1 ms; τ_2_ = 2 ms
Ff to Pyr	g^max^ = 0.055; E_N_ = −10 mV; τ_1_ = 4 ms; τ_2_ = 8 ms
Pyr to Fb	g^max^ = 0.25/0.06^†^; E_N_ = +70 mV; τ_1_ = 1 ms; τ_2_ = 2 ms
Fb to Pyr	g^max^ = 0.55; E_N_ = −10 mV; τ_1_ = 4 ms; τ_2_ = 8 ms
Pyr to Pyr (association fibers)	g^max^ = 510/260^†^; E_N_ = +70 mV; τ_1_ = 1 ms; τ_2_ = 2 ms
Pyr adaptation	A^ahc^ = 40/0^*^; E_N_ = −15 mV; τ^ahc^ = 100 ms

* *spiking neurons*.

† *different values are without/with NE modulation, respectively*.

‡ *The two Mi compartments are electrically coupled and the output computed in the apical compartment is directly applied to the soma compartment*.

We implemented the PC subnetwork with three cell types: pyramidal (Pyr) cells, feedforward interneurons (Ff), and feedback interneurons (Fb) (Suzuki and Bekkers, 2007; Stokes and Isaacson, 2010), with details as presented in (de Almeida et al., 2013; Devore et al., 2014) and parameters listed in Table 1. The present model contains 100 neurons of each cell type. In our previous work, we took care to adjust parameters for connectivity and neural responses to odorants to best match experimentally reported data. We chose to create networks of 100 neurons of each type as a compromise between simulation speed and enough neurons to allow statistical validity. The architecture and parameters in the model, other than those related to the function of NE investigated here, are kept similar to those used before to ensure continuity between models with the goal of a model capable of simulating different aspects of olfactory function. Details about connectivity can be found in Figure 1 and all neural and synaptic parameters are detailed in Table 1.

### Implementation

***Neurons*** are implemented as single compartment integrate-and-fire neurons, with the exception of Mi cells, which have two compartments (de Almeida et al., 2013). A first-order differential equation describes membrane voltage in a given compartment with respect to time (Hasselmo et al., 1997; Linster and Cleland, 2002; Linster et al., 2011):

(1) $$\tau \frac{dv(t)}{dt}+v(t)=V^{ext}(t)$$

where τ is the membrane time constant and *V^*ext*^*(*t*) is the external input over time. (see Table 1 for a comprehensive list of parameters). The two compartments of Mi cells are electrically coupled. As a consequence, Equation (1) is modified in Mi cells such that the difference in voltage between the apical compartment representing the apical dendrite and the soma compartment representing soma and lateral dendrites is fed directly into the soma compartment. This aligns with the physiological role of the Mi soma as an electrical integration site (Chen et al., 2002).

The external input *V^*ext*^* from a given presynaptic neuron is described by Equation (2):

(2) $$V_{j}^{ext}(t)=W_{ij}g_{ij}(t)\left[E_{N,ij}-v_{j}(t)\right]$$

where *W_ij_* is the strength of the synapse connecting neurons *i* and *j*, *g_i_*(*t*) is the conductance change in neuron *i* at time *t*, *E_Nij_* is the Nernst potential of the specific channel type, and *v_j_*(*t*) is the membrane potential of the postsynaptic neuron *j* at time *t*.

Depending on the type of neuron, network activity arises either through continuous output (modeling the average activity of a larger population in the case of OSNs or dendritic voltages in the case of PG cells), or by discrete spikes (for all other cell types). Equation (3) governs continuous output in the former case and instantaneous spiking probability in the latter case:

(3) $$F_{i}(V)=\{\begin{array}{ccc} 0 & \text{if} & V\leq \theta^{\min}\\ {\left(\frac{V-\theta^{\min}}{\theta^{\max}-\theta^{\min}}\right)}^{\beta } & \text{if} & \theta^{\max}<V<\theta^{\min}\\ 1 & \text{if} & V\geq \theta^{\max} \end{array}$$

where θ^*min*^ is the minimum firing threshold, θ^*max*^ is the saturation value, and β is a constant defining the nonlinearity of *Fi(v)*. For continuous presynaptic cells the conductance change *g_i_*(*t*) = *g*^max*^ F_*i*_[v(t)], while for spiking neurons *g_i_*(t) = *c*(*t* – *t*^*fire*^_*i*_), where *t*^*fire*^_*i*_ is the time of neuron's last spike. The conductance time course is described by Equation (4):

(4) $$g(t)=g^{\max}\left(e^{-t/\tau 1}-e^{-t/t2}\right)$$

where the dimensionless constant *g^max^* represents the maximum conductance of a given channel and τ*1* and τ*2* are the rise and fall times, respectively, of the conductance. A spiking neuron is reset to the hyperpolarization potential *v^*hyper*^* following an action potential, after which it remains inactive for its refractory period *t^*refrac*^*. See Table 1 for a comprehensive list of parameters used in these simulations.

***Neuronal adaptation*** was implemented in Pyr cells as a hyperpolarizing current that increases the firing threshold for recently active neurons. The conductance changes of the afterhyperpolarization current *V*^*ahc*^_*i*_ (*t*) in Pyr cell *i* are described in Equation (5):

(5) $$\tau^{ahc}\frac{dV_{i}^{ahc}}{dt}+V_{i}^{ahc}=A^{ahc}X_{i}$$

where *X_i_* is equal to 1 in the time-step after neuron *i* spikes and 0 otherwise. Therefore, *V^*ahc*^* increases with the constant *A^*ahc*^* and decays with the characteristic time τ^*ahc*^.

The effects of ***NE modulation*** in the OB are increasingly well characterized by experimental data (for review see Linster et al., 2011). These data show that noradrenergic activation of α1 receptors on mitral cells and α1 and α2 receptors on granule cells modulate overall activation. In mitral cells, α1 receptor activation can enhance responsiveness to weak inputs (Jiang et al., 1996; Ciombor et al., 1999; Hayar et al., 2001). At the same time, α1 activation increases granule cell excitability along with the strength of the inhibitory input from granule to mitral cells, while α2 activation has the opposite effect (Nai et al., 2009, 2010; Pandipati et al., 2010). As a result, at low NE concentrations, where α2 receptors are predominantly active because of differences in receptor affinities, mitral cell responses increase. At high NE concentrations, α1 receptor effects dominate α2 receptor effects, driving mitral cell activation toward baseline levels (Nai et al., 2009, 2010; Escanilla et al., 2010, 2012; Linster et al., 2011). Overall, mitral cell activation in the bulb appears to be mediated by a non-linear, dose-dependent response to NE in granule and mitral cells.

Cortical effects of NE include the overall suppression of feedback excitation through multiple mechanisms: suppression of inhibitory interneuron response to pyramidal cell excitation (Doze et al., 1991), direct depolarization of inhibitory interneurons (Gellman and Aghajanian, 1993; Marek and Aghajanian, 1996), suppression of autoassociative excitation between pyramidal cells (Hasselmo et al., 1997) and suppression of Pyr adaptation.

In our network, modulation by NE affects Mi and Gr cells in the OB and Pyr and Fb cells in the PC. The relationship between NE concentration and level of modulation *O* for the receptor type *i* is defined by Equation (6):

(6) $$O_{i}=\frac{1}{1+\left(\frac{Y_{i}}{C}\right)}$$

here, *Y* is the activation at which half-maximal modulation would be achieved (see Table 1 for details) and *C* is the NE concentration.

The graph in Figure 1Bi shows how NE concentration affects Gr spontaneous firing through α1 and α2 receptors, modeled after Nai et al. (2010). The effects of NE are modeled by changing the firing threshold parameter [θ^*min*^ (Equation (3))] on Gr neurons (Figure 1Bi), which results in changes in baseline activation. Figure 1Bii shows how NE affects Mi saturation threshold [represented by θ^*max*^ in Equation (3)], effectively rendering the Mi cells more excitable without changing baseline activity. Mi spontaneous firing is not affected, but responses to low stimuli are potentiated by the low firing threshold (Linster et al., 2011). Figure 1Biii shows NE modulation of cortical model cells: NE acts on Pyr cells in different ways: it suppresses excitatory synaptic transmission in autoassociative connections between Pyr neurons (Hasselmo et al., 1997), while suppressing firing adaptation. The suppression in autoassociative connections is implemented by reducing the efficiency of these synapses (Pyr-Pyr *G^max^* in Table 1) to 60% of its original value when NE modulation is high, while the suppression of adaptation is achieved by reducing the amplitude of the after hyperpolarization current [*A^*ahc*^* in Table 1; Equation (5)], rendering the cells less sensitive to hyperpolarization induced by previous spikes. In Fb interneurons, high NE reduces the efficiency from Pyr inputs (Doze et al., 1991), implemented in the model by a decrease in Pyr-Fb *G^max^* (Table 1) and increases spontaneous activity by reducing the value of θ^*min*^ [Equation (3)] on Fb neurons (Doze et al., 1991; Gellman and Aghajanian, 1993). NE concentration as represented in the model corresponds to experimental dosages ranging from 10–2 μM to 1 M. See Table 1 for a full list of parameters affected by NE modulation.

***Synaptic plasticity*** in Pyr cell associative connections are implemented in our network as Hebbian learning, where the synaptic strength *W* will be enhanced if both pre and postsynaptic neurons fire together, as shown in Equation (7):

(7) $$\frac{dW_{ij}^{syn}}{dt}-1-W_{ij}^{syn}\frac{i^{post}\left(t-t_{j}^{fire}\right)b^{glu}(t-t^{fire}-t^{delay})}{\tau^{pp}}$$

here, *W*^*syn*^_*ij*_ is the synaptic weight between neurons *i* and *j* while *t^*delay*^* is the time it takes for the action potential to travel from the soma to the recurrent connections. *i^*post*^* describes the evolution of the postsynaptic depolarization and *b^*glu*^* is the time course of the binding of glutamate on NMDA receptors. Changes in synaptic enforcement have been long attributed to the coactivation of these mechanisms (Bliss and Collingridge, 1993). Thus, in our model, if *i^*post*^* and *b^*glu*^* peak close to each other, *W*^*syn*^_*ij*_ is driven to one with characteristic time pp (800 ms). The time course of *i^*post*^* is described by Equation (8):

(8) $$i^{post}(t)=\frac{t}{\tau^{post}}\text{exp}\left(1-\frac{t}{\tau^{post}}\right)$$

where τ^*post*^ is the characteristic time of the depolarization at the postsynaptic neuron (2 ms). Finally, the binding of glutamate on NMDA receptors is described in Equation (9):

(9) $$b^{glu}(t)=\text{exp}\left(-\frac{t}{\tau^{NMDAf}}\right)\left(\text{exp}\left(1-\frac{t}{\tau^{NMDAr}}\right)\right)$$

here, τ^*NMDA*f^ = 7 ms and τ^*NMDAr*^ = 1 ms characterize the receptors' kinetics.

The weights of active associative synapses are initially set to a random value between 0 and 0.04, which is ~10% of the maximum weights after 4 five s training sessions. These weights are then normalized to a *W*' so that $\sum_{n\;=\;1}^{j}\sum_{m\;=\;1}^{j}{W^{\prime }}_{ij}=1$ and used in Equation (4). The Hebbian rule described in Equation (8) doesn't have unlearning, therefore the synaptic weights can only increase over training sessions. However, the synaptic strength between neurons that are part of the odor pattern are going to increase faster. The normalization helps to concentrate the synaptic increments among this group connections.

An ***odorant*** is represented by its receptor affinity values across all OSNs, where affinity correlates with strength of activation in a given OSN. Odorants are generated through a randomly permuted array of 100 different affinity values corresponding to the number of OSNs in the model, with affinity values computed from a normal probability density function *N*(*x*, μ, σ) with *x* in the range (1, 100), μ = 50, and σ = 10. Thus, any generated odorant elicits an equal average response, given equal odorant concentrations. Odorant concentration varies between 0 and a max-concentration value of 1, the saturation point for OSN activation. For ease of visualization, raster plots are centered around glomerulus 50.

All simulations were implemented using the MATLAB programming language using the Euler integration method for differential equations with a time step of 0.5 ms. This model can be accessed from the modelDB website (Hines et al., 2004) at the link senselab.med.yale.edu/ModelDb/ accession number 146813. The significance of the result in **Figures 5**–**7** were corrected for multiple comparisons using the Bonferroni method.

### Analysis

The most basic level of feature analysis in our model is measuring **average firing frequencies** in populations of neurons. The figures presented here focus on responses of cell types modulated by NE. In the OB, these include Gr and Mi cells; in the PC; these include Fb interneurons and Pyr cells. Neural activity in response to odorants stabilizes quickly (100–200 ms); because we create a novel instantiation of the network and odor stimulations for every data point, we chose a 1000 ms window to average firing rates. This ensures that randomization and noise do not unduly influence the results.

The **detection index** defined here measures how distant the activity evoked by an odor stimulation is from spontaneous activity. We first calculate the “baseline distance,” defined as the average Euclidean distance between two simulations of spontaneous activity only. We consider detection as an Euclidean distance higher than the average ± two standard deviations of the baseline distance. Levels of detection are therefore calculated by subtracting the average baseline distance and then dividing by two times the standard deviation from the calculated Euclidean distance between spontaneous activity and odor evoked activity: indices above 1 are considered detectable by the network.

The sparseness of weight distribution described in **Figure 7** is defined by Equation (10):

(10) $$S=\frac{1\left(\frac{{\left(\sum_{i\;=\;1}^{N}\frac{W_{i}}{N}\right)}^{2}}{\sum_{i\;=\;1}^{N}\frac{W_{i}}{N^{2}}}\right)}{1-\frac{1}{N}}$$

where *W_i_* is the active synaptic weight *i* and *N* is the total active synapses. A response is highly sparse (*S* = 1) when a single synapse concentrates all the weight, while it has minimal sparseness (*S* = 0) when we have an equal weight distribution among all synapses.

## Results

### Bulbar and cortical NE modulate odor detection

**Figure 2:**
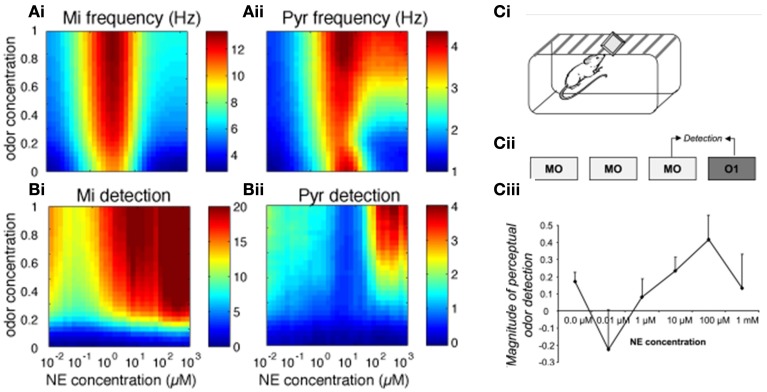
**Bulbar and cortical NE modulate odor detection**. The graphs show how different levels of NE modulation affect firing rates and detection for a range of odor concentrations. The colormaps show 30 × 30 grid of firing rate and detection values as a function of NE levels (x-axis) and odor concentration (y-axis), with warmer colors representing higher amplitudes. **(A)** Mi and Pyr firing rates; **(B)** Mi and Pyr odor detection. **(C)** Related behavioral results, reprinted from Linster et al. (2011). **(C_i_, C_ii_)** Spontaneous odor detection task. Rats were presented with mineral oil or odors diluted in mineral oil placed on a weighing dish on the lid of the home-cage and investigation times were recorded **(Ci)**. Three presentations of mineral oil (MO) were followed by one presentation of an odorant (O1) diluted to approximate vapor partial pressure of 10^−6^ Pa **(C_ii_)**. The graph on **(C_iii_)** shows the magnitude of perceptual detection as a function of dosage of NE infusions into the OB. The magnitude of detection was calculated as the difference between the response to the last MO trial and the odor (O1) trial divided by their sum.

We first investigated how bulbar and cortical NE modulate odor detection over a range of NE and odor concentrations. The color maps in Figure 2 show how changes in NE (modeled to match experimental data, see Figure 1; x-axis) and odor concentration (y-axis) affect bulbar and cortical firing rates and odor detection. Both bulbar and cortical spontaneous activity (odor concentration = 0) are strongly modulated by NE in a non-uniform manner (Figure 2A). The non-linearity arises from differential effects of NE on α1 and α2 receptors in the OB (Nai et al., 2009, 2010; Figure 1B), first decreasing inhibition due to α2 receptor activation, and then increasing inhibition due to α1 receptor activation, paired with increased excitation of mitral cells at medium NE levels. At high NE levels the increased inhibition overrides excitability and the result is a net decrease in mitral cell activity (Figure 2Ai, warmer colors mean higher firing rates). This non-uniform effect on spontaneous firing rates is mimicked in cortical pyramidal cells (Figure 2Aii), which in the naive state is driven almost exclusively by bulbar inputs. As odor stimulation increases, mitral cell firing rates increase but are still highly dependent on NE modulation. Pyramidal cell activity reflects inputs from the OB except when both odor stimulation and NE are high and the increased excitability of pyramidal cells in response to odorants influences cortical response more than bulbar inputs (Figure 2Aii). Odor detection (see methods) is highly modulated by NE in OB and PC and interestingly does not follow the same non-uniform distribution as observed for firing rates (Figure 2B, warmer colors mean better detection; a detection value > 1 means that the network can just detect the odorant). As shown behaviorally, detection slightly decreases and then increases with NE concentration (Figure 2C). In the PC, detection is overall weaker except for very high odor NE and odor concentrations (Figure 2Bii). Figure 2C shows how behavioral modulation of NE levels in the OB compares to the simulation results (reprinted from Linster et al., 2011). Briefly, rats were habituated to mineral oil (MO), the carrier that odorants are diluted in, during three 1 min trials separated by 5 min intertrial intervals (Figure 2Ci). During the fourth trial, rats were presented with an odorant (O1) diluted to a very low, subthreshold concentration corresponding to a vapor partial pressure of 10^−6^ Pa. Detection of this odorant was assessed by comparing the investigation times during the last mineral oil trial (MO) to that in response to the odorant (O1) and the magnitude of detection was calculated as the difference between these two trials divided by their sum. The graph in Figure 2Cii shows the magnitude of odor detection as a function of the dosage of NE infused into the OBs 20 min before the behavioral session. This graph can be qualitatively compared to detection levels of mitral cells (Figure 2Bi) at low odor concentrations (0.4 to 0.5 of maximum concentration in the model).

**Figure 3:**
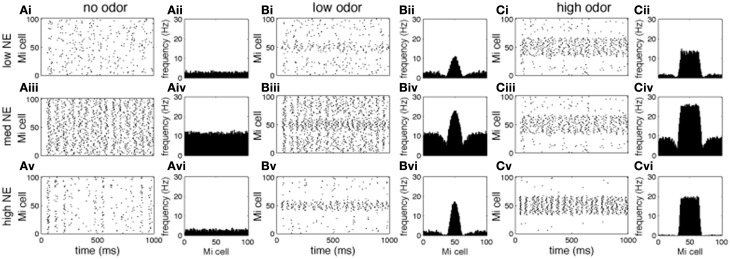
**Mi cell odor responses**. Each graph in the figure shows either a raster plot of Mi cell firing during a 1 s simulation or the average firing rate over a 10 s simulation. In these tests the most active cells in the odor patterns are centered around neuron 50, to facilitate visualization. This is not a reflection of the physical arrangement of cells in the model. **(A)** Mi spontaneous activity with low [**(A_i_)**, corresponding to 10^−2^ uM in Figure 2], medium (**A_ii_**, 1 uM), and high (**A_iii_**, 1 M) NE modulation. **(B)** Mi responses to low concentration odorants (c = 0.2) with low **(B_i_)**, medium **(B_ii_)**, and high **(B_iii_)** NE modulation. **(C)** Mi responses to high concentration odorants (c = 0.8) with low **(A_i_)**, medium **(A_ii_)**, and high **(A_iii_)** NE modulation.

**Figure 4:**
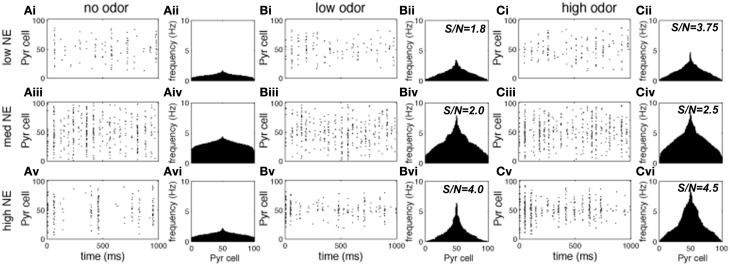
**Pyr cell odor responses**. Each graph in the figure shows either a raster plot of Pyr cell firing during a 1 s simulation or the average firing rate over a 10 s simulation. In these tests the most active cells in the odor patterns are centered around neuron 50, to facilitate visualization. This is not a reflection of the physical arrangement of cells in the model. **(A)** Pyr spontaneous activity with low (10^−2^ uM; **A_i_**), medium (1 uM; **A_ii_**) and high (1 M; **A_iii_**) NE modulation. **(B)** Pyr responses to low concentration (c = 0.2) odorants with low **(B_i_)**, medium **(B_ii_)**, and high **(B_iii_)** NE modulation. **(C)** Pyr responses to high concentration (c = 0.8) odorants with low (10^−2^ uM; **A_i_**), medium (1 uM; **A_ii_**), and high (1 M; **A_iii_**) NE modulation.

Figures 3, 4 show examples of the neural activities underlying these phenomena; the graphs show rasterplots and spike histograms of bulbar and cortical activities as a function of odor concentration and NE levels. Note that for ease of visualization, odor responsive cells have been artificially grouped together in these graphs, this is not a representation of the physical organization of these cells in the model. Figure 3A illustrates how spontaneous activity increases then decreases as NE concentration is increased in the OB; this effect arises from the differential affinities of α1 and α2 receptors (Figure 1B). Figure 3B clarifies why mitral cell firing rates and odor detection do not simply co-vary: levels of spontaneous activity and odor evoked responses are independently regulated by NE to a certain degree. Activity in PC (Figure 4) seems to mostly follow that observed in Mi cells with the exception that generally detection is lower because odor responses are less pronounced and more distributed in a naïve cortical network.

### Role of bulbar and cortical NE for odor detection

**Figure 5:**
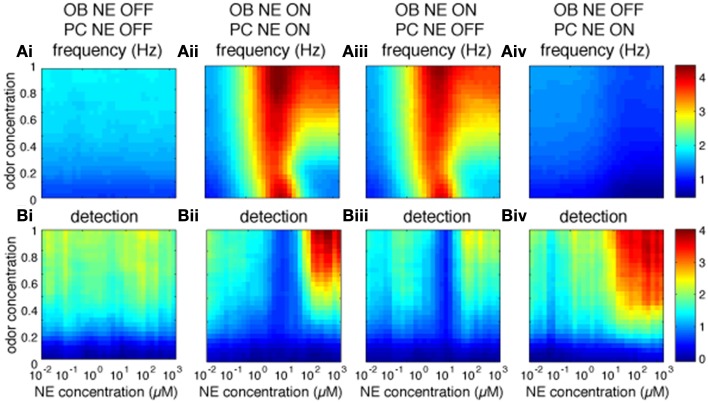
**Role of bulbar and cortical NE for odor detection**. The graphs show how different levels of NE modulation in OB and PC separately affect PC firing rates and detection for a range of odor concentrations. The colormaps show 30 × 30 grid of firing rate **(A)** and detection values **(B)** in PC pyramidal cells as a function of NE levels (x-axis) and odor concentration (y-axis), with warmer colors representing higher amplitudes. **(A_i_,B_i_)** show Pyr firing rates and detection when NE modulation is OFF in both OB and PC. **(A_ii_,B_ii_)** show control simulations with NE modulation ON in both OB and PC. **(A_iii_,B_iii_)** show results for NE modulation ON in OB only and **(A_iv_,B_iv_)** for NE modulation ON in PC only. Blocking NE in both OB and PC has a significant effect on firing rates as compared to control (*P* < 0.001, using Wilcoxon signed rank test; as has blockade of OB or PC NE (*P* < 0.001). In contrast, blocking NE in PC only has no significant effect on firing rates (*P* = 0.46), suggesting that cortical NE modulation plays a small role in our simulations.

The OB is thought to pre-process odor representations to enable and modulate cortical learning depending on behavioral demands (Doucette et al., 2011; Devore et al., 2014). Most behavioral experiments to date have locally blocked NE modulation in the OB and observed perceptual and learning effects which may well be mediated by cortical rather than bulbar processes (Mandairon et al., 2008; Doucette et al., 2007; Escanilla et al., 2010; Linster et al., 2011). It is therefore important to understand what role bulbar modulation plays in cortical processing. Figure 5 shows to what extend cortical processing is modulated by bulbar NE (Figure 5A: Pyr firing rates and Figure 5B: cortical detection, warmer colors mean higher firing rates or detection). Both firing rates and detection in PC are decreased when all NE modulation is off (Figures 5Ai,Bi) as compared to all on (Figures 5Aii,Bii). Cortical NE by itself does not affect cortical firing rates (compare Figures 5Aii,Aiii), however, cortical detection is somewhat decreased (compare Figures 5Bii,Biii). Cortical NE alone in the absence of bulbar NE leads to very low firing rates whereas detection is less affected (Figures 5Aiv,Biv); these results suggest an important role in odor detection for cortical NE. Simulations shown in Figures 5Aii–iv are significantly different than control (Figure 5Ai; *p* < 0.001).

The graphs in Figure 5B show the effects of bulbar or cortical NE on detection levels. Levels of detection for all configurations (Figures 5Aii–iv) are significantly different from the control version (Figure 5A_i_; *P* < 0.001) with no NE modulation at all. Interestingly, the difference between NE active in OB and PC (Figure 5Bii) or only OB (Figure 5Biii) is not significant, suggesting that NE modulation in our model PC plays a important role in odor detection.

In summary, the simulations suggest that NE modulation in both OB and PC might play an important role in Pyr activity patterns.

### The impact of NE on cortical learning

**Figure 6:**
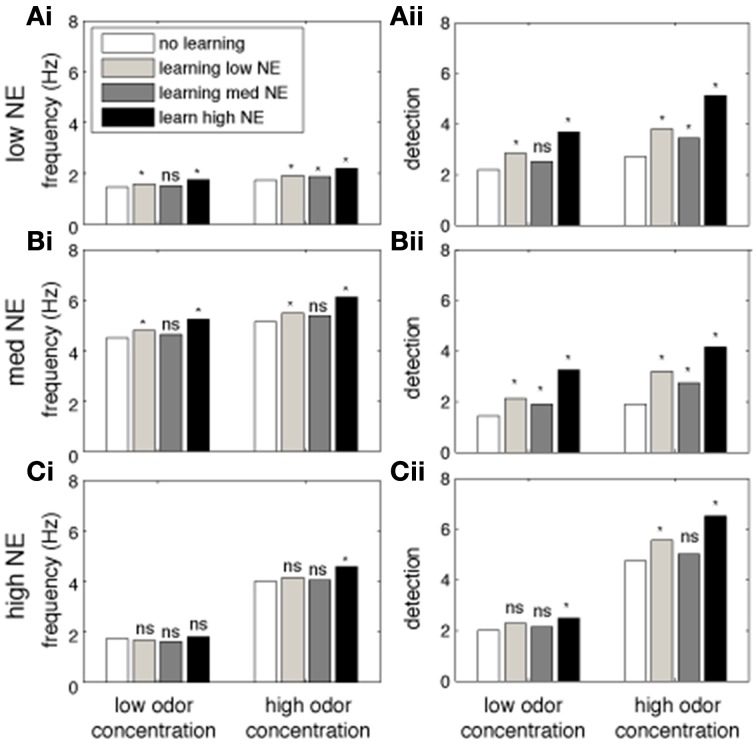
**The impact of NE on cortical learning**. The graphs show the effects of training the Pyr network at different levels of NE modulation on recall of the trained odor. Each bar shows the average measurement over 40 tests using randomly chosen odorants. The white bars show tests where no learning (training) was performed. Light gray bars show responses after learning with low NE (10^−2^ μM). Dark gray bars show recall after learning with medium NE (1 μM) and black bars used high (1 M) levels of NE. Bars on the left show recall of low concentration (*c* = 0.2) odorants, on the right of high concentration (*c* = 0.8) odorants. Recall under low (10^−2^ uM), medium (1 uM) and high (1M) concentrations of NE is shown in **(A–C)**, respectively. The PC network was first trained on a randomly chosen odor for 4 consecutive 5 s training sessions; the same odorant is then presented during the recall step and average firing rates and detection are calculated. Recall responses are compared to initial responses with no training (^*^indicates *p* < 0.001; student *t*-test).

Cortical associative learning is thought to be an important part of odor processing and long term memory. In our cortical model, learning is mediated through activity dependent plasticity on cortical association fibers, as first suggested by Haberly and Bower (1989) and studied in more detail by Hasselmo and colleagues (Hasselmo et al., 1992; Hasselmo and Bower, 1993). Briefly, during cortical learning, pyramidal cells responding to the presented odor strengthen synapses between them. Eventually pyramidal cell activity is driven by association fibers more than by bulbar input and activity reflects learned intrinsic information rather than afferent input only. We have previously shown that bulbar processing strongly affects cortical read out and learning of olfactory information, as well as the quality of the formed memory (de Almeida et al., 2013). We tested how cortical learning affects odor detection and how the presence of NE in the system modulates the learning and recall of odors. To do this, cortical networks were trained on odorants under three different levels of NE in OB and PC (low, medium and high, corresponding to 10^−2^, 1 and 1 M NE), using two concentrations of odorants (low and high, corresponding to 0.2 and 0.8 of the maximum possible odor concentration). After learning, odors were then recalled (at the same concentration) under low, medium and high NE. Recall simply means that the system is stimulated with a more or less noisy representation of a previously learned odorant and cortical responses to these odorants are recorded. Comparing these post-learning responses to pre-learning responses allows to assess to what degree learning changes odor representations in PC. The graphs in Figure 6 show cortical firing rates and detection during recall (after learning with different levels of NE). Each graph shows data for one level of NE during recall in a naïve network (no learning) or networks trained with low, medium, or high NE. We first observe that in the absence of learning, in agreement with previous simulations, a non-linear relationship exists between NE levels and firing rates and detection in the PC (white bars in Figures 6A,B). Overall, high levels of NE during learning are beneficial for later recall, independently of NE during recall (black bars in graphs A, B, and C). Interestingly, high levels of NE during recall are detrimental for the recall of low odor concentrations, with a smaller effect at higher odor concentrations (Figures 6Ci,Cii). It is interesting to note that high NE levels are beneficial during learning but not recall, specifically for detection of low concentration odorants. This is probably due to the suppression of excitatory synaptic transmission within the cortical network which interferes with recall of learned associative memories but is beneficial for the formation of these memories (Hasselmo et al., 1997).

**Figure 7:**
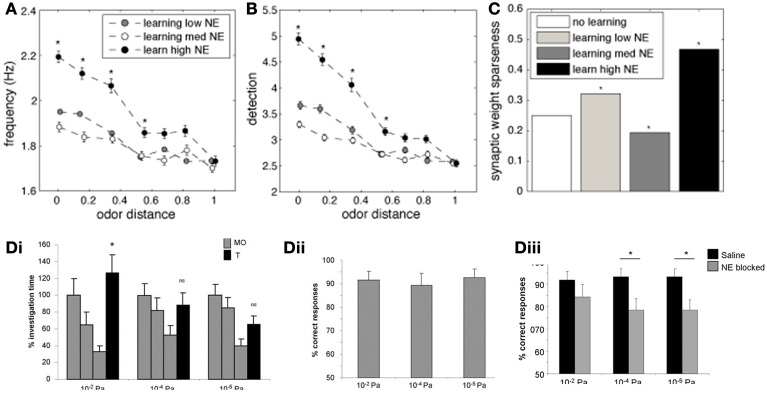
**The impact of NE on cortical associative memory performance**. Simulations presented here show recall performance when odors are distorted or perturbed with respect to the learned representation. Degree of perturbation is indicated by the normalized Euclidean distance between the recalled and the learned odor (x-axis). **(A)** Pyr firing rates as a function of degree of perturbation and NE levels during learning. **(B)** Cortical detection levels as a function of degree of perturbation and NE levels during learning. **(C)** Sparseness of learned synaptic weights as a function of learning and NE levels. (**D**) Behavioral results showing that reward-driven odor detection has substantially lower detection thresholds **(D_ii_)** than spontaneous detection **(Di)** and that the difference in thresholds depends on functioning NE receptors [reprinted from Escanilla et al. (2012)]. The graphs in **(D_i_)** show average investigation times in response to three presentations of mineral oil (MO) and one presentation of odorant (O1) at 10^−6^, 10^−4^, and 10^−2^ Pa. Note that rats only detected 10^−2^ Pa odorants, as indicated by a significant increase in investigation time. **(D_ii_)** The graph shows the percent correct choice made during 20 trials during which a diluted odorant was rewarded in the presence of a second pot containing unrewarded MO. Note that rats were able to detect odorants as low as 10^−6^ Pa when encouraged to do so with a food reward. **(D_iii_)** Detection of low concentration odorants in the forced choice task was impaired at 10^−6^ and 10^−4^ Pa when NE receptors were blocked during the behavioral task.

An important measure for cortical learning is how robust a learned representation is to perturbations of the original pattern. To test this, we compare the average firing frequency and levels of detection when recalling a simulated odorant that differs from the learned representation (odor distance in Figures 7A,B). The similarity between the learned and recalled odorants is calculated as the Euclidean distance between the input stimuli (at OSN levels): low odor distances indicate activity patters that are very similar to each other (small perturbations). The odorants where trained using either low (10^−2^ μM, white circles), medium (1 μM, gray circles), or high (1 M, black circles) NE concentration, but always recalled at low NE concentration.

Figure 7A shows the average firing frequency in response to odorants based on their similarity with the previously learned odor pattern. When the similarity between trained and recalled odorant is high (low distance), the average firing rate evoked is high, since the network can rely on the learned autoassociative connections of Pyr network rather than only on bulbar inputs for recall. The higher NE during the learning process, the higher the recall frequency. Odor detection levels show recall properties similar to firing rates (Figure 7B). Interestingly, when recall odorants exceed a certain distance from the learned odor (> 0.4), levels of NE during learning do not affect recall anymore; in general, the less perturbed the odor pattern, the more influence NE during learning has on recall. This suggests that high NE creates stronger and more robust attractors of learned representations.

Figure 7C shows how different levels of NE concentration during training affect weight distribution among Pyr autoassociative connections. A low sparseness indicates a less successful learning process because synaptic weights are less differentiated and odor specific. As suggested by the measures shown in Figures 7A,B, the learning process is most efficient at high NE levels. This result is probably due to the fact that both mitral and pyramidal cell firing rates increase, leading to faster learning, while increased inhibition in PC leads to sparser Pyr responses.

Behaviorally, we have shown that rats can quickly learn to detect odorant concentrations not spontaneously detected when motivated by a food reward (Escanilla et al., 2012). Figure 7D_i_ shows investigation times during the spontaneous discrimination test described in Figure 2C (reprinted from Escanilla et al., 2012). Rats decrease their investigation times to mineral oil over the course of three presentations (MO); when subsequently presented with an odorant at near threshold concentration (O1), they investigate odorants at vapor partial pressures of 10^−2^ Pa significantly more than mineral oil. Odorants at concentrations lower than 10^−2^ Pa were not treated differently from mineral oil, suggesting that these were not spontaneously detected. In contrast, when rats were trained to retrieve a reward in a scented dish paired with an unscented dish (mineral oil only), they could efficiently learn to detect odorants as low as vapor partial pressures of 10^−6^ Pa. The graph in Figure 7Dii shows the percent correct choices made over the course of 20 trials for odors diluted to approximate vapor partial pressures of 10^−2^, 10^−4^, and 10^−6^ Pa. These results are in agreement with the simulations presented: training improves the detection capabilities of said network. The performance of rats on very low odor concentrations was reduced when NE receptors were blocked (Figure 7Diii), leaving only those concentrations also detected spontaneously untouched. These behavioral results suggest that NE modulation is important for cortical learning, as suggested by our simulations. In summary, reward driven learning improves odor detection and is dependent on functioning NE levels.

## Discussion

Noradrenaline is deeply integrated into the olfactory system. Olfactory cues increase the discharge of LC neurons in behaving animals (Aston-Jones and Bloom, 1981) and trigger rapid increases in NE levels in OB as well as the accessory olfactory bulb (Kaba and Keverne, 1988; Kaba et al., 1989; Brennan et al., 1990).

Recently, increased attention has been paid by several labs to noradrenergic modulation of main olfactory bulb processing in adult animals (Doucette et al., 2007; Shea et al., 2008; Nai et al., 2009, 2010; Escanilla et al., 2010, 2012; Linster et al., 2011); we here add to these efforts by showing how noradrenergic modulation in olfactory bulb and cortex cooperate to enhance the detection and learning of low concentration odorants.

In our computational model of the olfactory bulb, NE modulation affects the detection of low concentration odorants in a non-uniform manner via dose-dependent changes of inhibitory tone and mitral cell excitability. These changes reflect modulation of perceptual odor detection seen in behavioral studies (Figure 2; Escanilla et al., 2010; Linster et al., 2011) and shed insight onto the neural mechanisms underlying behavioral observations. Figure 2C shows how our simulations are qualitatively comparable to the behaviorally observed changes in odor detection threshold with increasing infusions of NE into the OB. The observed non-linearities (decreased detection at low NE concentration followed by improved detection at higher concentration) probably result from the differential affinities of α1 and α2 receptors reported *in vitro* (Nai et al., 2009, 2010). Low NE concentrations activate mainly α2 receptors, leading to inhibition of inhibitory neurons, and as a consequence spontaneous activity is increased while mitral cell excitability is not yet modulated. As NE concentrations rise, α1 and β receptors are recruited, granule cell excitation overrides inhibition, and mitral cells are rendered more excitable. These modulatory effects lead to lower spontaneous rates accompanied by stronger odor responses, lowering overall detection thresholds. Changes in odor detection could be observed in both OB and PC in our model, but detailed simulations showed that these are mainly carried by modulation of OB responses imposed onto olfactory cortex. Overall, the effects on spontaneous odor detection seen in these computational models reflect the effects seen behaviorally when NE was manipulated in the olfactory bulb alone.

As evidenced by our model, cortical NE modulation becomes relevant to odor processing to a higher degree once cortical learning is included in the simulations. While we did not exhaustively explore the role of NE on cortical learning here, but rather implemented known effects on cortical processing, our results show that learning under high NE modulation improves cortical odor representations, whereas recall is impaired by high NE modulation. Bulbar NE strongly modulates the input to cortical cells, and therefore is one of the factors determining odor responses in a naïve cortical network. After the learning process, bulbar input only partially drives cortical representations. These are now dominated by cortical association fibers strengthened through the learning process. Figure 6 shows that in the model, NE during learning enhances the process, presumably because of increased excitation and decreased afterhyperpolarization of cortical pyramidal cells; such a net increase in activity would enhance the degree of synaptic plasticity between pyramidal cells (even it the plasticity rule itself is not changed). Recall is hindered by NE due to the suppression of association fiber transmission by noradrenergic input: with high NE, association fiber transmission is suppressed and cortical representations become dominated by bulbar inputs again. Behaviorally, our lab and others have observed that blocking NE receptors impairs the initial acquisition but not the asymptotic learning in a reward-associative task, suggesting a role for learning but not recall (Mandairon et al., 2008; Doucette et al., 2007; Escanilla et al., 2012). Given that our behavioral results show a predominant role for NE in processing of low amplitude odors, one could envision that NE inputs to the OB and cortex are regulated at least partially by activity in these same systems. Putatively, such a regulation can happen locally by presynaptic regulation of transmitter release (Gervais, 1987). Figure 7C shows that odor detection thresholds are significantly decreased by reward—associative learning and that this effect is dependent on functioning NE receptors. We find in the model that cortical learning improves odor detection at very low concentrations, suggesting a possible neural mechanism for this behavioral effect.

Computational modeling can explore the effects of modulators in qualitative ways, we here attempt to draw comparisons between NE effects measured *in vitro* and behavioral observations. It is likely that NE levels fluctuate around a baseline imposed by low spontaneous rates of LC neurons during awake states, making it difficult to draw qualitative comparisons between brain slice data, modeling and behavioral pharmacology. In summary, our model suggests two functions of modulation by noradrenaline based on the location of action. NE released in the OB appears to modulate the representations of an odor in both bulbar mitral cells and cortical pyramidal cells in a naïve cortex. On the other hand, NE released in the cortex seems to mainly modulate associative learning.

### Conflict of interest statement

The authors declare that the research was conducted in the absence of any commercial or financial relationships that could be construed as a potential conflict of interest.
